# Spontaneous Resolution of Cystic Adventitial Disease of the Popliteal Artery

**DOI:** 10.1155/2021/8863682

**Published:** 2021-03-10

**Authors:** Georges Ibrahim, Sami Nabhani, Michel Feghaly, Georges Baaklini

**Affiliations:** Division of Vascular Surgery, Saint Georges Hospital, University Medical Center, Ashrafieh, Beirut, Lebanon

## Abstract

Spontaneous resolution of cystic adventitial disease (CAD) is rare with occasional reports in the literature. In this case report, we are describing a 30-year-old man who presented with rapid onset of severe intermittent claudication and was diagnosed with CAD. Resection of the lesion with autologous vein replacement was scheduled. However, the claudication suddenly improved at 4 weeks after onset. Ultrasonography and computed tomography revealed regression of the cystic lesions with resolution of the popliteal artery stenosis. His symptoms did not recur during the 12-month follow-up period. Although it is unclear whether this resolution is permanent, in this report, we describe our experience with a case of CAD that eventually spontaneously regressed and the possibility of conservative treatment.

## 1. Introduction

Cystic adventitial disease (CAD) of the popliteal artery is an uncommon cause of intermittent claudication in young patients. Several treatment options are available, oriented to either drainage of the cyst and/or arterial reconstruction. Endovascular techniques have been used in exceptional cases to treat this condition, with mixed to poor results. Reports of spontaneous resolution of the disease are rare. We report our experience with a case of CAD that eventually spontaneously regressed.

## 2. Case Report

**Figure 1:**
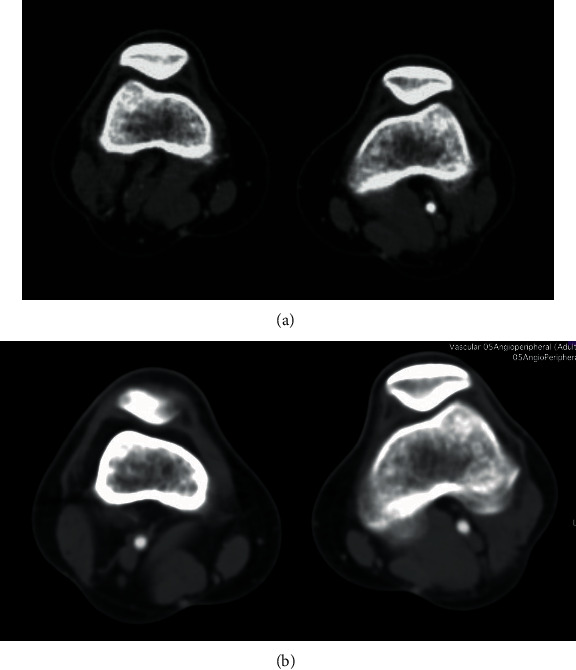
Transverse cut of the CT angiography at the level of the knee: (a) at time of presentation with the arrow showing an obstruction in the right popliteal artery and (b) done at 6 months postpresentation.

**Figure 2:**
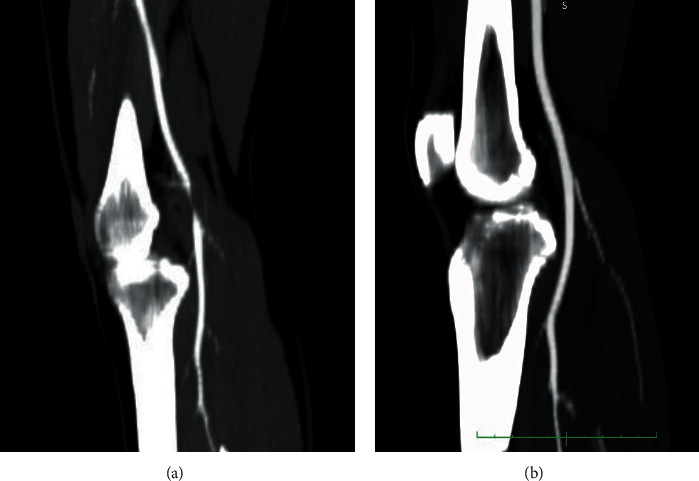
Saggital cut of the CT angiography at the level of the knee: (a) at time of presentation with the arrow showing an obstruction in the right popliteal artery and (b) done at 6 months postpresentation, with no sign of obstruction.

A 30-year-old male presented to our hospital for rapid onset of severe claudication after walking 100 meters. History of smoking was his only cardiovascular risk factor. On examination, he had a cold right foot with no pulses palpable below the femoral artery on that side, with normal examination on the contralateral limb. Duplex scan (DS) of the lower limb arteries followed by contrast enhanced computed tomography (CT) revealed a cystic lesion in the right popliteal artery with severe narrowing of the right popliteal artery lumen (Figures 1(a) and 2(a)).

The patient was therefore diagnosed with cystic adventitial disease and was scheduled to undergo resection with vein replacement surgery.

The surgery was delayed due to scheduling issues. However, claudication suddenly improved at 2 months after initial presentation. Examination showed normal distal pulses, and duplex scan was repeated and showed normal flow in the right popliteal artery with no sign of cystic compression. We therefore cancelled the surgery and decided on outpatient follow-up.

Contrast enhanced CT was done at 6 months postinitial presentation and revealed that the CAD had disappeared with no signs of right popliteal artery stenosis (Figures 1(b) and 2(b)).

**Figure 3:**
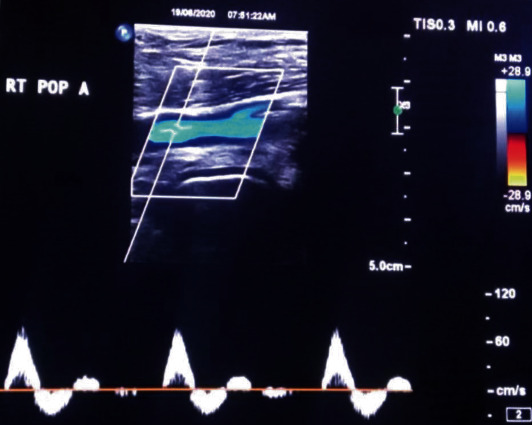
Duplex scan of the right popliteal artery at 12-month follow-up, showing normal triphasic flow.

At the 12-month follow-up, the patient was still asymptomatic with no changes in the physical examination or on the DS done (Figure 3). The patient continues to be followed up as outpatient.

Full consent from the patient was obtained for publishing this article and images.

## 3. Discussion

CAD typically appears in healthy male patients in their fourth to fifth decades of life. The most common presentation is unilateral claudication secondary to popliteal artery stenosis or occlusion. CAD has been reported in the external iliac artery, brachial, radial, and ulnar arteries as well as the saphenous vein around the ankle area. All cysts are para-articular, suggestive of an association between CAD formation and its respective neighboring joint [1, 2].

Etiology of the disease is still unclear with many speculations. The ganglion theory states that adventitial cysts are formed by capsular synovial structures growing and tracking in the adventitia along vascular branches. This is supported by the fact that the cysts are similar to the ganglions morphologically, with high concentration of hyaluronic acid [3]. The development theory states that mucin secreting mesenchymal cells from the adjacent joint are entrapped in the adventitia of the developing artery [4].

Treatment options are mainly excision of the diseased artery with interposition grafting, excision of the cyst with preservation of the artery, and percutaneous drainage of the cysts or endovascular treatment with balloon angioplasty, with and without stenting [1].

Most disease recurrences occurred after percutaneous aspiration and angioplasty. That is why we originally opted for cyst excision and bypass, as it is the most favorable option within the current literature. Spontaneous regression of CAD of the popliteal artery has been previously reported in 9 cases [5–12], with some claiming permanent resolution of the disease after 10 years of follow-up [8], while others had recurrence of symptoms with the eventual need for a more definitive treatment [13].

In cases of spontaneous regression of CAD with few or no significant symptoms, it is reasonable to opt for regular outpatient follow-up focusing on history and physical examination with or without imaging modalities.

## 4. Conclusion

We have described a rare case of spontaneous regression of CAD. Long-term follow-up is reasonable due to the risk of recurrence. In case of relapse, surgery can always be planned to ensure durable result.

## Data Availability

Further data concerning the case can be requested through email.
